# Supplementary material for: Ionizing radiation responses appear incidental to desiccation responses in the bdelloid rotifer Adineta vaga
Source: BMC Biol. 2024 Jan 25;22:11. doi: 10.1186/s12915-023-01807-8 (PMC10809525; doi:10.1186/s12915-023-01807-8)
Supplement: Supplementary file 1 — Additional file 1: Fig S1. Survival and fertility rates of A. vaga individuals exposed to desiccation and radiation stress. Percentage of survival (dark blue) and fertility rate of A. vaga exposed to desiccation, rehydration post 14 days desiccation, and 500 Gy of X-rays (low-LET) or Fe-ions (high-LET). The fertility is represented by three different histograms: fraction of individuals able to produce viable offspring (green), fraction of individuals unable to produce viable offspring but only sterile egg(s) (blue), fraction of individuals unable to lay egg or with premature dead (red). Survival rate was evaluated 2 days post rehydration or post radiation. To ensure reliable results, survival data were obtained from at least three replicates. Samples exposed to 500 Gy of iron ions were transported from Belgium to GSI and then returned to the authors' laboratory for analysis. To evaluate the impact of transportation on the samples, a control group labeled "Ctl transport" was also sent to GSI. This control group experienced similar conditions as the exposed samples but did not undergo radiation exposure. Fig S2. Volcano plots of differential genes over-expressed (OE) and under-expressed (UE) in A) A. vaga individuals entering desiccation, B) 2.5 h post desiccation and x-rays radiation. The percentage of the genome over- and under-expressed are written. Although a higher percentage of genes are under-expressed in B) the log2foldchange values are bigger in OE than UE genes. Fig S3. Number of genes with GO ids for a specific log2foldchange values identified as A) over-expressed (OE) genes in A. vaga rotifers entering desiccation, B) under-expressed (UE) genes in A. vaga rotifers entering desiccation, C) 906 OE genes in the core response to radiation 2.5 h post irradiation, D) 724 OE genes in the core response to irradiation 8 h post radiation. Fig S4. Gene Ontology enrichment analyses GO biological processes significantly enriched (chi-square test p-value < 0.05, min. 3 [file 12915_2023_1807_MOESM1_ESM.zip › Fig_S4_GO_enrichment_supple.pdf]

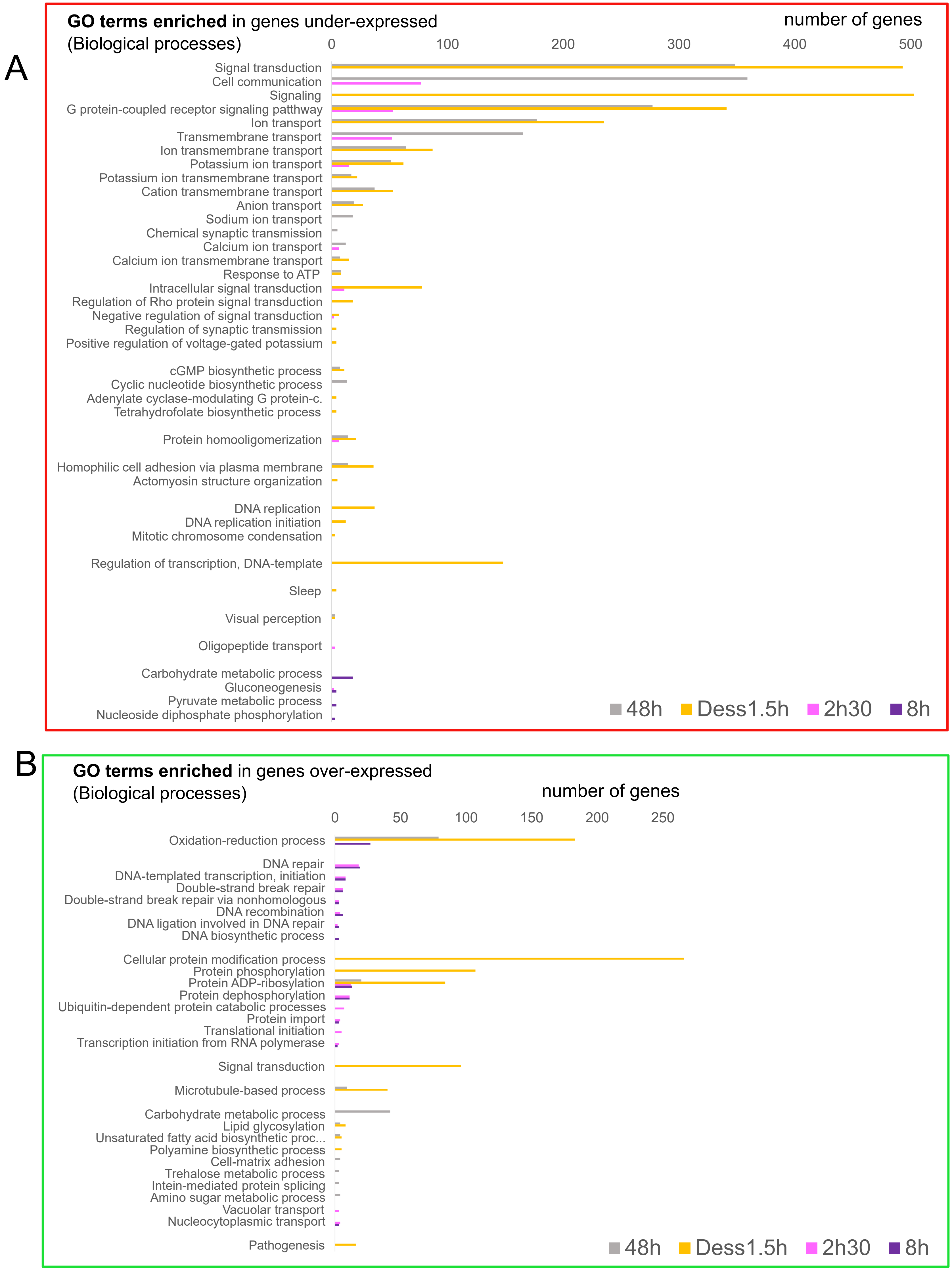
A
GO terms enriched in genes under-expressed
(Biological processes)
number of genes
0
100
200
300
400
500
Signal transduction
Cell communication
Signaling
G protein-coupled receptor signaling patthway
Ion transport
Transmembrane transport
Ion transmembrane transport
Potassium ion transport
Potassium ion transmembrane transport
Cation transmembrane transport
Anion transport
Sodium ion transport
Chemical synaptic transmission
Calcium ion transport
Calcium ion transmembrane transport
Response to ATP
Intracellular signal transduction
Regulation of Rho protein signal transduction
Negative regulation of signal transduction
Regulation of synaptic transmission
Positive regulation of voltage-gated potassium
cGMP biosynthetic process
Cyclic nucleotide biosynthetic process
Adenylate cyclase-modulating G protein-c.
Tetrahydrofolate biosynthetic process
Protein homooligomerization
Homophilic cell adhesion via plasma membrane
Actomyosin structure organization
DNA replication
DNA replication initiation
Mitotic chromosome condensation
Regulation of transcription, DNA-template
Sleep
Visual perception
Oligopeptide transport
Carbohydrate metabolic process
Gluconeogenesis
Pyruvate metabolic process
Nucleoside diphosphate phosphorylation
48h
Dess1.5h
2h30
8h
B
GO terms enriched in genes over-expressed
(Biological processes)
number of genes
0
50
100
150
200
250
Oxidation-reduction process
DNA repair
DNA-templated transcription, initiation
Double-strand break repair
Double-strand break repair via nonhomologous
DNA recombination
DNA ligation involved in DNA repair
DNA biosynthetic process
Cellular protein modification process
Protein phosphorylation
Protein ADP-ribosylation
Protein dephosphorylation
Ubiquitin-dependent protein catabolic processes
Protein import
Translational initiation
Transcription initiation from RNA polymerase
Signal transduction
Microtubule-based process
Carbohydrate metabolic process
Lipid glycosylation
Unsaturated fatty acid biosynthetic proc...
Polyamine biosynthetic process
Cell-matrix adhesion
Trehalose metabolic process
Intein-mediated protein splicing
Amino sugar metabolic process
Vacuolar transport
Nucleocytoplasmic transport
Pathogenesis
48h
Dess1.5h
2h30
8h
