# Supplementary figures and images for: Ionizing radiation responses appear incidental to desiccation responses in the bdelloid rotifer Adineta vaga
Source: BMC Biol. 2024 Jan 25;22:11. doi: 10.1186/s12915-023-01807-8 (PMC10809525; doi:10.1186/s12915-023-01807-8)

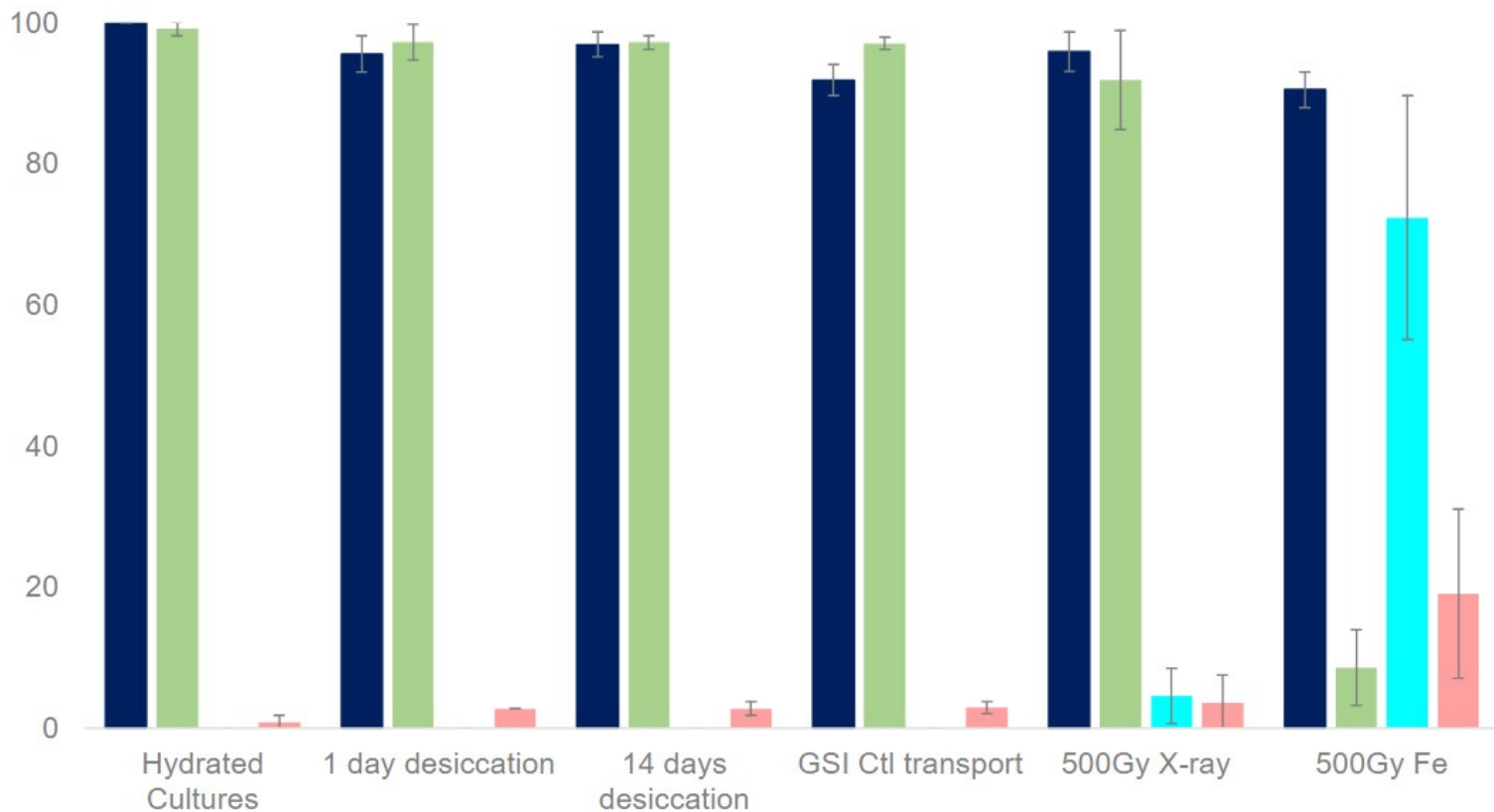

Supplement: Supplementary file 1 — Additional file 1: Fig S1. Survival and fertility rates of A. vaga individuals exposed to desiccation and radiation stress. Percentage of survival (dark blue) and fertility rate of A. vaga exposed to desiccation, rehydration post 14 days desiccation, and 500 Gy of X-rays (low-LET) or Fe-ions (high-LET). The fertility is represented by three different histograms: fraction of individuals able to produce viable offspring (green), fraction of individuals unable to produce viable offspring but only sterile egg(s) (blue), fraction of individuals unable to lay egg or with premature dead (red). Survival rate was evaluated 2 days post rehydration or post radiation. To ensure reliable results, survival data were obtained from at least three replicates. Samples exposed to 500 Gy of iron ions were transported from Belgium to GSI and then returned to the authors' laboratory for analysis. To evaluate the impact of transportation on the samples, a control group labeled "Ctl transport" was also sent to GSI. This control group experienced similar conditions as the exposed samples but did not undergo radiation exposure. Fig S2. Volcano plots of differential genes over-expressed (OE) and under-expressed (UE) in A) A. vaga individuals entering desiccation, B) 2.5 h post desiccation and x-rays radiation. The percentage of the genome over- and under-expressed are written. Although a higher percentage of genes are under-expressed in B) the log2foldchange values are bigger in OE than UE genes. Fig S3. Number of genes with GO ids for a specific log2foldchange values identified as A) over-expressed (OE) genes in A. vaga rotifers entering desiccation, B) under-expressed (UE) genes in A. vaga rotifers entering desiccation, C) 906 OE genes in the core response to radiation 2.5 h post irradiation, D) 724 OE genes in the core response to irradiation 8 h post radiation. Fig S4. Gene Ontology enrichment analyses GO biological processes significantly enriched (chi-square test p-value < 0.05, min. 3 [file 12915_2023_1807_MOESM1_ESM.zip › Fig_S1.pdf]

A

Desiccation entry

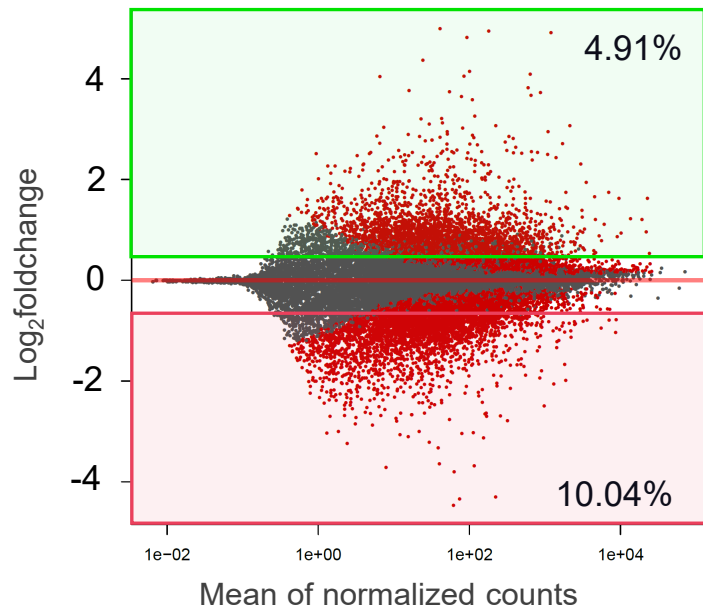

B

2.5 hours post desiccation+x-rays

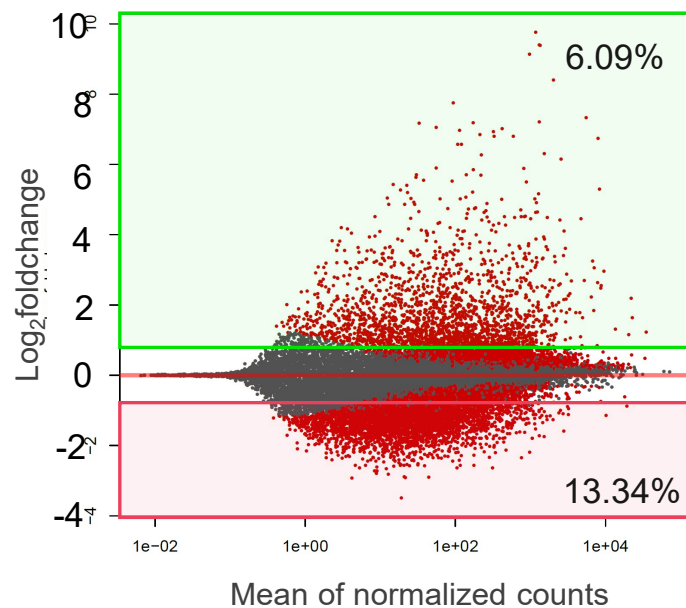

Supplement: Supplementary file 1 — Additional file 1: Fig S1. Survival and fertility rates of A. vaga individuals exposed to desiccation and radiation stress. Percentage of survival (dark blue) and fertility rate of A. vaga exposed to desiccation, rehydration post 14 days desiccation, and 500 Gy of X-rays (low-LET) or Fe-ions (high-LET). The fertility is represented by three different histograms: fraction of individuals able to produce viable offspring (green), fraction of individuals unable to produce viable offspring but only sterile egg(s) (blue), fraction of individuals unable to lay egg or with premature dead (red). Survival rate was evaluated 2 days post rehydration or post radiation. To ensure reliable results, survival data were obtained from at least three replicates. Samples exposed to 500 Gy of iron ions were transported from Belgium to GSI and then returned to the authors' laboratory for analysis. To evaluate the impact of transportation on the samples, a control group labeled "Ctl transport" was also sent to GSI. This control group experienced similar conditions as the exposed samples but did not undergo radiation exposure. Fig S2. Volcano plots of differential genes over-expressed (OE) and under-expressed (UE) in A) A. vaga individuals entering desiccation, B) 2.5 h post desiccation and x-rays radiation. The percentage of the genome over- and under-expressed are written. Although a higher percentage of genes are under-expressed in B) the log2foldchange values are bigger in OE than UE genes. Fig S3. Number of genes with GO ids for a specific log2foldchange values identified as A) over-expressed (OE) genes in A. vaga rotifers entering desiccation, B) under-expressed (UE) genes in A. vaga rotifers entering desiccation, C) 906 OE genes in the core response to radiation 2.5 h post irradiation, D) 724 OE genes in the core response to irradiation 8 h post radiation. Fig S4. Gene Ontology enrichment analyses GO biological processes significantly enriched (chi-square test p-value < 0.05, min. 3 [file 12915_2023_1807_MOESM1_ESM.zip › Fig_S2_volcano_plots.pdf]

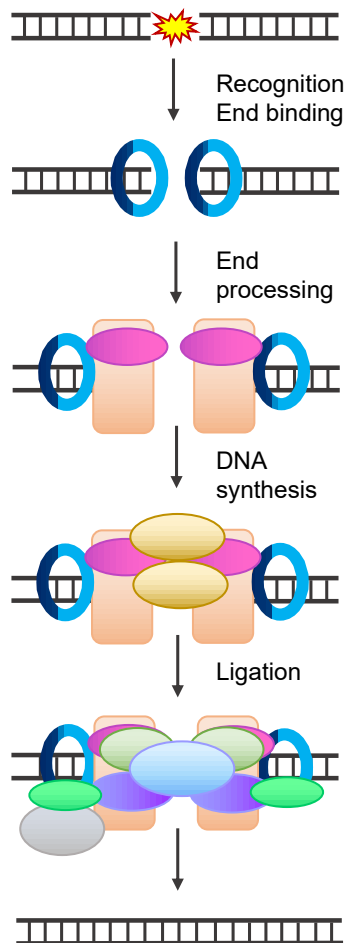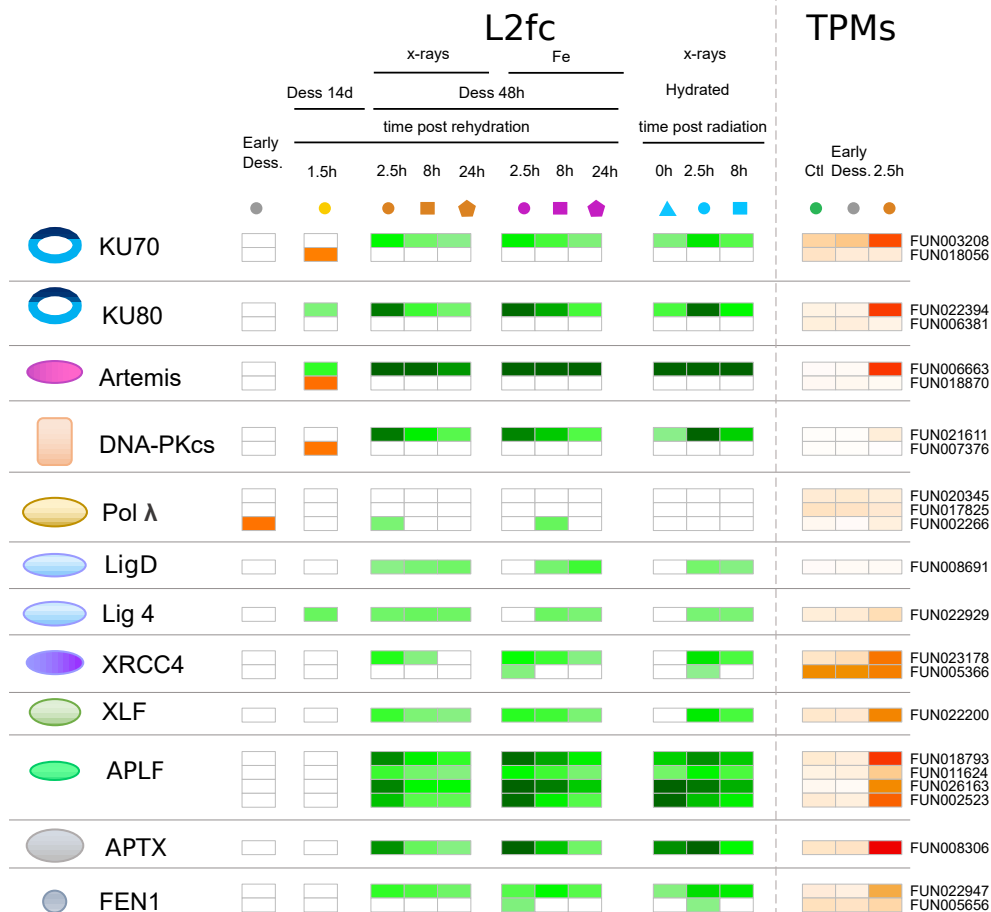

Supplement: Supplementary file 1 — Additional file 1: Fig S1. Survival and fertility rates of A. vaga individuals exposed to desiccation and radiation stress. Percentage of survival (dark blue) and fertility rate of A. vaga exposed to desiccation, rehydration post 14 days desiccation, and 500 Gy of X-rays (low-LET) or Fe-ions (high-LET). The fertility is represented by three different histograms: fraction of individuals able to produce viable offspring (green), fraction of individuals unable to produce viable offspring but only sterile egg(s) (blue), fraction of individuals unable to lay egg or with premature dead (red). Survival rate was evaluated 2 days post rehydration or post radiation. To ensure reliable results, survival data were obtained from at least three replicates. Samples exposed to 500 Gy of iron ions were transported from Belgium to GSI and then returned to the authors' laboratory for analysis. To evaluate the impact of transportation on the samples, a control group labeled "Ctl transport" was also sent to GSI. This control group experienced similar conditions as the exposed samples but did not undergo radiation exposure. Fig S2. Volcano plots of differential genes over-expressed (OE) and under-expressed (UE) in A) A. vaga individuals entering desiccation, B) 2.5 h post desiccation and x-rays radiation. The percentage of the genome over- and under-expressed are written. Although a higher percentage of genes are under-expressed in B) the log2foldchange values are bigger in OE than UE genes. Fig S3. Number of genes with GO ids for a specific log2foldchange values identified as A) over-expressed (OE) genes in A. vaga rotifers entering desiccation, B) under-expressed (UE) genes in A. vaga rotifers entering desiccation, C) 906 OE genes in the core response to radiation 2.5 h post irradiation, D) 724 OE genes in the core response to irradiation 8 h post radiation. Fig S4. Gene Ontology enrichment analyses GO biological processes significantly enriched (chi-square test p-value < 0.05, min. 3 [file 12915_2023_1807_MOESM1_ESM.zip › Fig_S5_NHEJ_new_format.pdf]

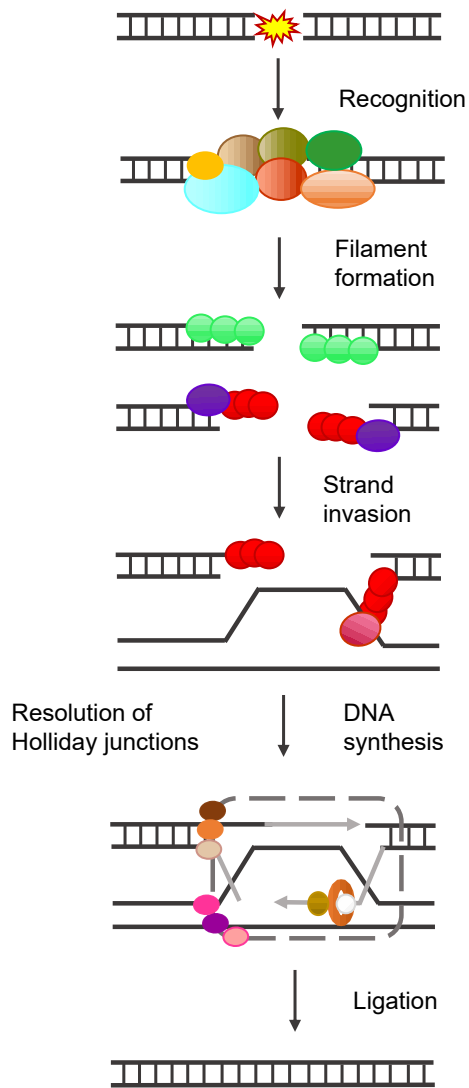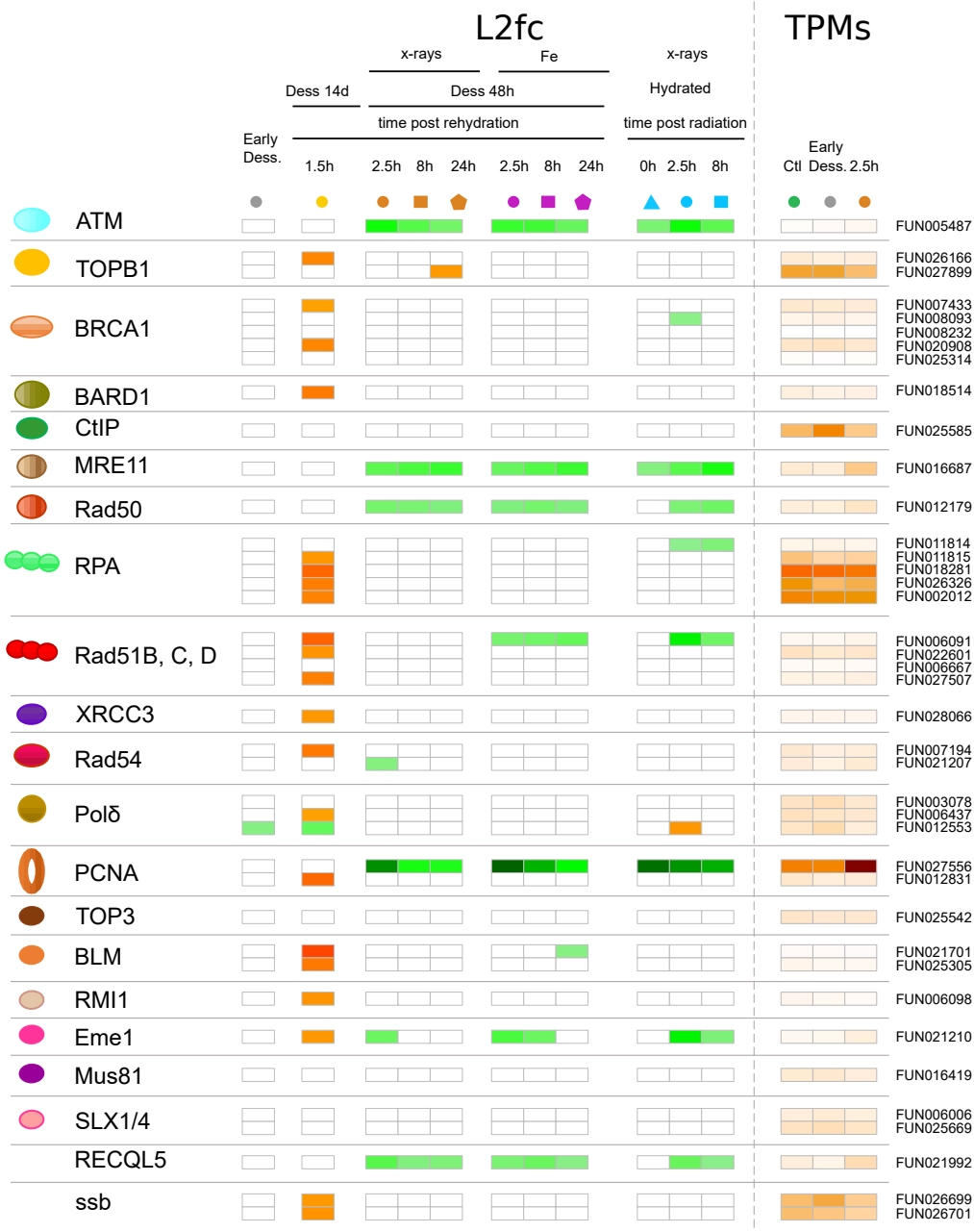

# Legend

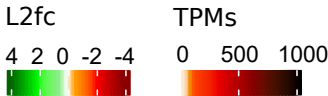

Supplement: Supplementary file 1 — Additional file 1: Fig S1. Survival and fertility rates of A. vaga individuals exposed to desiccation and radiation stress. Percentage of survival (dark blue) and fertility rate of A. vaga exposed to desiccation, rehydration post 14 days desiccation, and 500 Gy of X-rays (low-LET) or Fe-ions (high-LET). The fertility is represented by three different histograms: fraction of individuals able to produce viable offspring (green), fraction of individuals unable to produce viable offspring but only sterile egg(s) (blue), fraction of individuals unable to lay egg or with premature dead (red). Survival rate was evaluated 2 days post rehydration or post radiation. To ensure reliable results, survival data were obtained from at least three replicates. Samples exposed to 500 Gy of iron ions were transported from Belgium to GSI and then returned to the authors' laboratory for analysis. To evaluate the impact of transportation on the samples, a control group labeled "Ctl transport" was also sent to GSI. This control group experienced similar conditions as the exposed samples but did not undergo radiation exposure. Fig S2. Volcano plots of differential genes over-expressed (OE) and under-expressed (UE) in A) A. vaga individuals entering desiccation, B) 2.5 h post desiccation and x-rays radiation. The percentage of the genome over- and under-expressed are written. Although a higher percentage of genes are under-expressed in B) the log2foldchange values are bigger in OE than UE genes. Fig S3. Number of genes with GO ids for a specific log2foldchange values identified as A) over-expressed (OE) genes in A. vaga rotifers entering desiccation, B) under-expressed (UE) genes in A. vaga rotifers entering desiccation, C) 906 OE genes in the core response to radiation 2.5 h post irradiation, D) 724 OE genes in the core response to irradiation 8 h post radiation. Fig S4. Gene Ontology enrichment analyses GO biological processes significantly enriched (chi-square test p-value < 0.05, min. 3 [file 12915_2023_1807_MOESM1_ESM.zip › Fig_S6_HR_new_heatmap_reformat_A4.pdf]

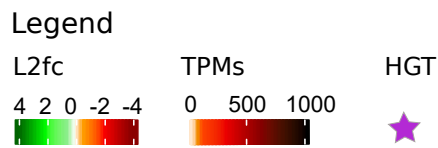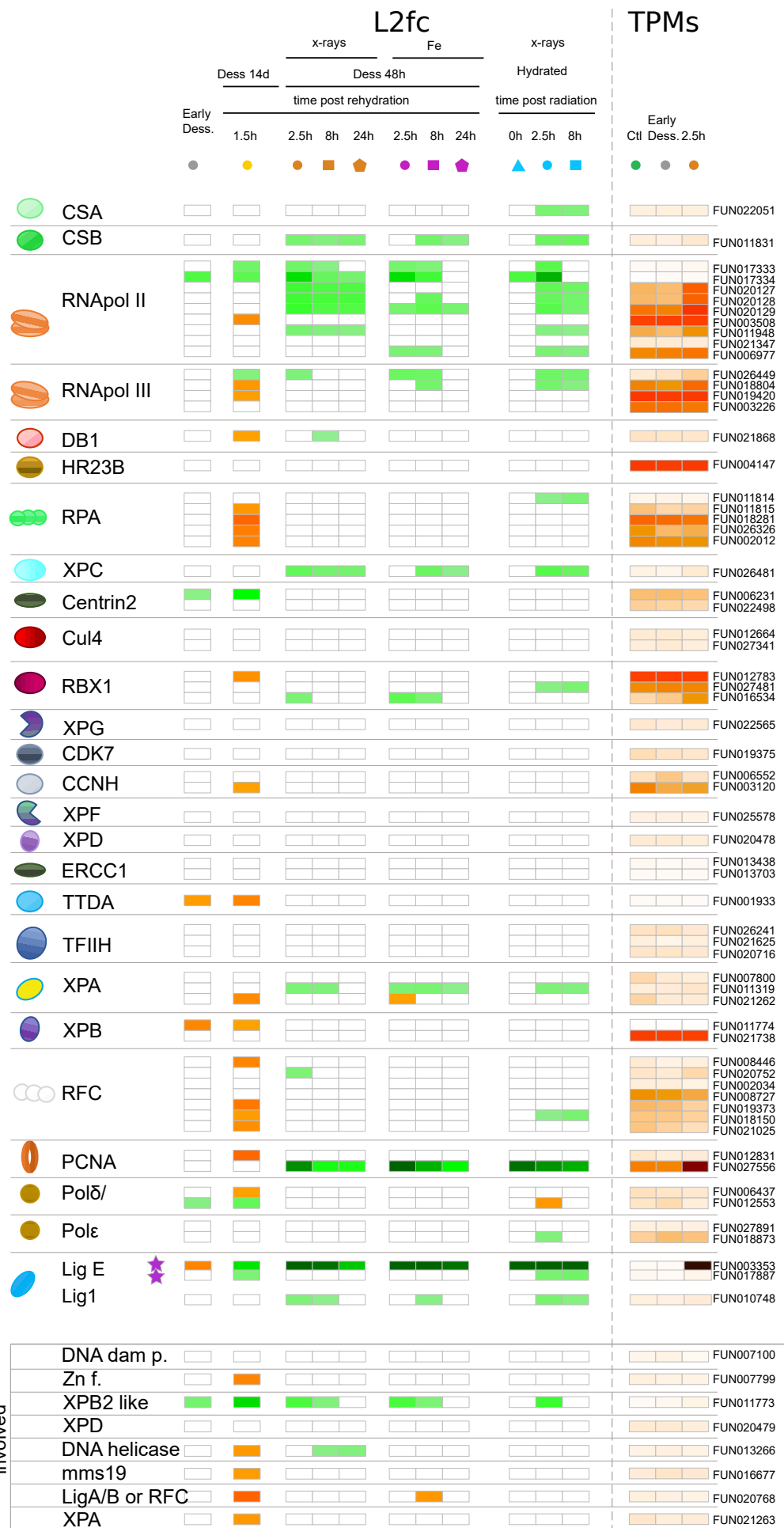

Supplement: Supplementary file 1 — Additional file 1: Fig S1. Survival and fertility rates of A. vaga individuals exposed to desiccation and radiation stress. Percentage of survival (dark blue) and fertility rate of A. vaga exposed to desiccation, rehydration post 14 days desiccation, and 500 Gy of X-rays (low-LET) or Fe-ions (high-LET). The fertility is represented by three different histograms: fraction of individuals able to produce viable offspring (green), fraction of individuals unable to produce viable offspring but only sterile egg(s) (blue), fraction of individuals unable to lay egg or with premature dead (red). Survival rate was evaluated 2 days post rehydration or post radiation. To ensure reliable results, survival data were obtained from at least three replicates. Samples exposed to 500 Gy of iron ions were transported from Belgium to GSI and then returned to the authors' laboratory for analysis. To evaluate the impact of transportation on the samples, a control group labeled "Ctl transport" was also sent to GSI. This control group experienced similar conditions as the exposed samples but did not undergo radiation exposure. Fig S2. Volcano plots of differential genes over-expressed (OE) and under-expressed (UE) in A) A. vaga individuals entering desiccation, B) 2.5 h post desiccation and x-rays radiation. The percentage of the genome over- and under-expressed are written. Although a higher percentage of genes are under-expressed in B) the log2foldchange values are bigger in OE than UE genes. Fig S3. Number of genes with GO ids for a specific log2foldchange values identified as A) over-expressed (OE) genes in A. vaga rotifers entering desiccation, B) under-expressed (UE) genes in A. vaga rotifers entering desiccation, C) 906 OE genes in the core response to radiation 2.5 h post irradiation, D) 724 OE genes in the core response to irradiation 8 h post radiation. Fig S4. Gene Ontology enrichment analyses GO biological processes significantly enriched (chi-square test p-value < 0.05, min. 3 [file 12915_2023_1807_MOESM1_ESM.zip › Fig_S7_NER_heatmap.pdf]

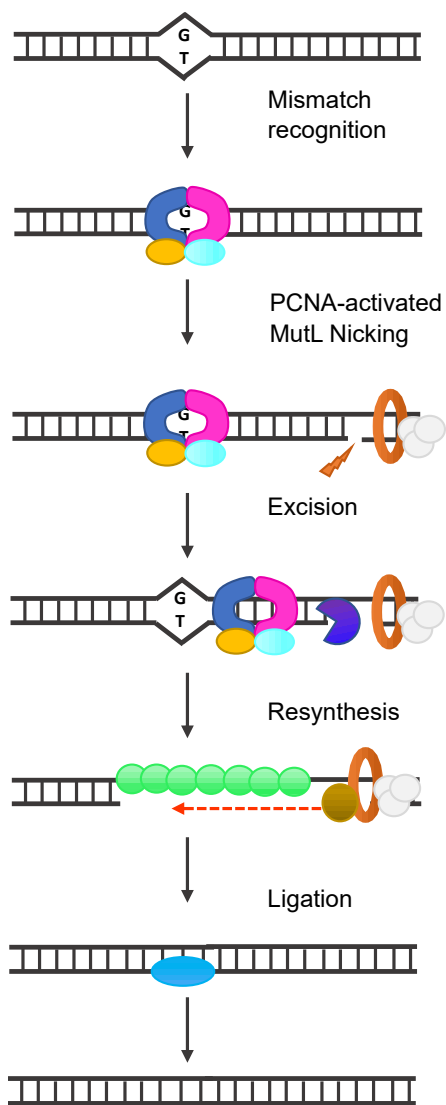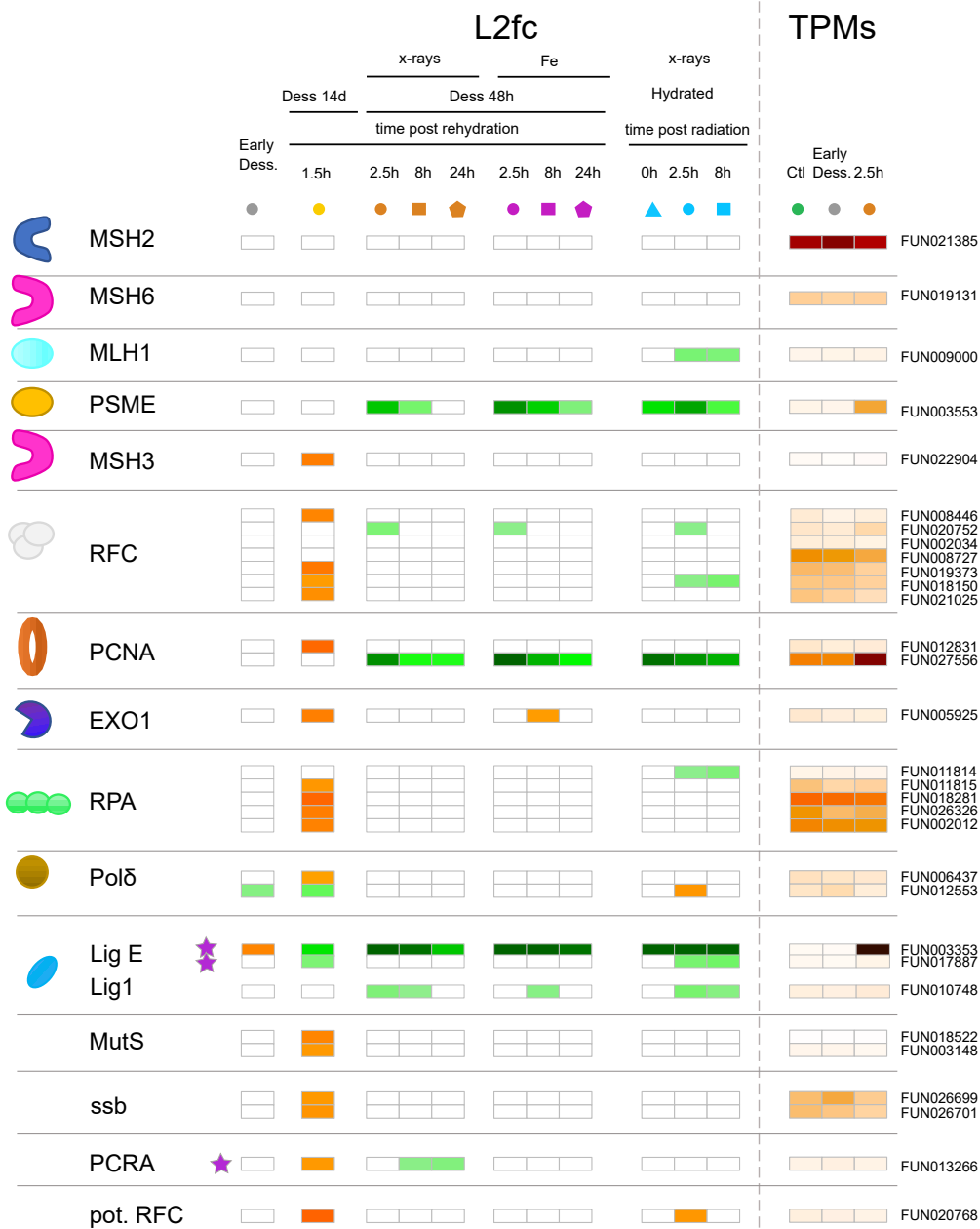

## Legend

L2fc

4 2 0 -2 -4

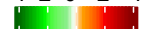

TPMs

0 500 1000

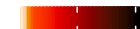

HGT

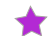

Supplement: Supplementary file 1 — Additional file 1: Fig S1. Survival and fertility rates of A. vaga individuals exposed to desiccation and radiation stress. Percentage of survival (dark blue) and fertility rate of A. vaga exposed to desiccation, rehydration post 14 days desiccation, and 500 Gy of X-rays (low-LET) or Fe-ions (high-LET). The fertility is represented by three different histograms: fraction of individuals able to produce viable offspring (green), fraction of individuals unable to produce viable offspring but only sterile egg(s) (blue), fraction of individuals unable to lay egg or with premature dead (red). Survival rate was evaluated 2 days post rehydration or post radiation. To ensure reliable results, survival data were obtained from at least three replicates. Samples exposed to 500 Gy of iron ions were transported from Belgium to GSI and then returned to the authors' laboratory for analysis. To evaluate the impact of transportation on the samples, a control group labeled "Ctl transport" was also sent to GSI. This control group experienced similar conditions as the exposed samples but did not undergo radiation exposure. Fig S2. Volcano plots of differential genes over-expressed (OE) and under-expressed (UE) in A) A. vaga individuals entering desiccation, B) 2.5 h post desiccation and x-rays radiation. The percentage of the genome over- and under-expressed are written. Although a higher percentage of genes are under-expressed in B) the log2foldchange values are bigger in OE than UE genes. Fig S3. Number of genes with GO ids for a specific log2foldchange values identified as A) over-expressed (OE) genes in A. vaga rotifers entering desiccation, B) under-expressed (UE) genes in A. vaga rotifers entering desiccation, C) 906 OE genes in the core response to radiation 2.5 h post irradiation, D) 724 OE genes in the core response to irradiation 8 h post radiation. Fig S4. Gene Ontology enrichment analyses GO biological processes significantly enriched (chi-square test p-value < 0.05, min. 3 [file 12915_2023_1807_MOESM1_ESM.zip › Fig_S8_MMR_format_A4_publication.pdf]
